# Comparison of the Efficacy of Intralesional Ascorbic Acid Mesotherapy and Intralesional Tranexamic Acid in Treating Melasma in the Skin of Colour Population

**DOI:** 10.7759/cureus.87851

**Published:** 2025-07-13

**Authors:** Hina Aslam, Anjum Muhammad, Asmat Ullah, Namrah Aziz, Jamil A Lakhiar, Bilal Ahmad, Atika Shahzadi, Adeel Ahmad, Chandni, Gurnam Virdi

**Affiliations:** 1 Pharmacology, King Edward Medical University, Lahore, PAK; 2 Dermatology, Combined Military Hospital, Kohat, PAK; 3 Healthcare Administration, Combined Military Hospital, Kohat, PAK; 4 Endocrinology and Diabetes, Combined Military Hospital, Kohat, PAK; 5 Forensic Medicine, Combined Military Hospital, Kohat, PAK; 6 Family Medicine, Combined Military Hospital, Kohat, PAK; 7 General Medicine, Combined Military Hospital, Kohat, PAK; 8 Dermatology, Academy of Aesthetic Medicine, Sheffield, GBR

**Keywords:** ascorbic acid, cutaneous hyperpigmentation, intralesional tranexamic acid, melasma, melasma area severity index, mesotherapy, skin of colour

## Abstract

Background: Melasma remains an important dermatological ailment affecting diverse types of skin across the world. Various treatment modalities are currently employed to control melasma; however, the ideal treatment remains elusive. Comparative data regarding the efficacy of intralesional tranexamic acid (TA) and ascorbic acid (AA) mesotherapy (MT) in the treatment of melasma in South Asian skin is sparse. The objective of this study was to compare the clinical efficacy and safety of intralesional tranexamic acid versus ascorbic acid mesotherapy for treating melasma in patients with skin of colour (SOC).

Methodology: This quasi-experimental study was conducted at the Dermatology Department of Combined Military Hospital Kohat, a tertiary healthcare setup in Pakistan, between April 16, 2024, and October 16, 2024. A total of 110 volunteer patients with melasma, aged 18 to 55 years, were included. Individuals with a history of other dermatological conditions such as cutaneous infections, discoid lupus erythematosus, positive pregnancy tests, lactation, oral contraceptive use, or anaemia were excluded from the research. The patients were randomly assigned to two groups. In Group A, 55 patients were included and administered fortnightly sessions of intralesional TA MT, while in Group B, 55 patients received fortnightly sessions of intralesional AA MT. Both groups were also prescribed a daily Kligman regimen-based cream at night, along with sun protection measures in the form of broad-spectrum SPF 60 creams. Final evaluation was done at the end of 12 weeks, during which the Melasma Area Severity Index (MASI) was calculated and compared. The side effect profiles in both groups were also recorded.

Results: Of the total 110 patients, around two-thirds (n = 77, 70%) were found to have mixed-type melasma, while the rest (n = 33, 30%) presented with dermal melasma. The average age of patients in Group A was 34.4 ± 5.1 years, while in Group B, it was 32.8 ± 4.5 years. Around two-thirds (n = 79, 71.8%) of the cohort were in the age bracket of 18-35 years. The efficacy of intralesional TA MT (Group A) was found to be higher than that of intralesional AA MT (Group B) when modified MASI scores were compared. For TA, the baseline MASI ranged from 6.4 to 8.1 (mean 7.2), and end-treatment MASI ranged from 3.3 to 5.6 (mean 4.45), showing a clear reduction of 2.75 in the mean MASI score. For AA, the baseline MASI ranged from 6.3 to 7.9 (mean 7.1), and end-treatment MASI ranged from 5.8 to 6.7 (mean 6.25), showing a clear reduction of 0.85 in the mean MASI score. The difference was statistically significant, with a p-value of 0.001. Intralesional TA MT was also associated with a lower incidence of irritation compared to intralesional AA MT, although this difference was not statistically significant.

Conclusion: The use of intralesional TA MT in melasma in South Asian skin appears more effective in clearing melasma than intralesional AA MT. It also remains a cheaper and safer treatment option for the control of melasma. Further large-scale randomised controlled studies are warranted to ascertain these findings.

## Introduction

Melasma is a chronic pigmentary skin disorder characterised by dark brown patches on the skin, most commonly affecting the photo-exposed sites of the face [1]. It afflicts scores of individuals across the globe, with a much higher prevalence in women and individuals with darker skin tones [2]. There is a far higher incidence of melasma in females than in males (female-to-male ratio of about 4:1), particularly in multiparous women, where around 51% of pregnant women are affected as compared to 25% of unmarried females [3].

Despite its seemingly benign nature, melasma has the potential to substantially impact the quality of life of affected individuals due to its aesthetic and psychosocial implications. The exact cause of melasma remains a mystery; however, various pathophysiological factors have been proposed as underlying causes. These include solar UV light-mediated overproduction of melanin, hormonal influences during pregnancy, use of oral contraceptives, hormone replacement treatments, certain medications, and a positive family history [4-6].

The treatment strategies currently employed for melasma remain far from perfect, as they mainly focus on controlling pigmentation. The search for an optimal treatment strategy is still ongoing. Commonly used management options include sun protection creams or lotions, elimination of aggravating factors, and various topical agents such as hydroquinone (HQ), retinoids, alpha- and beta-hydroxy acids like kojic and salicylic acid, and corticosteroids. These medications are used in different combinations with equally variable success rates in melasma patients [3,7].

There is growing interest in the use of mesotherapy (MT), in which intradermal injections are administered into melasma lesions using various agents, such as tranexamic acid (TA) and ascorbic acid (AA; vitamin C), with variable outcomes [8-10].

While mesotherapy with both TA and AA might seem like rational options for the treatment of melasma, there is a limited amount of data on the comparative efficacy of MT using TA and AA, particularly in the skin of colour (SOC) population. Another important factor for dermatologists working in countries with SOC populations is a special need to opt for strategies that are both cost-effective and efficacious in treating melasma, particularly in resource-limited settings where patients cannot afford costly and prolonged interventions.

The aim of this study was to compare the efficacy and safety of MT using TA and AA in the treatment of melasma in the SOC population.

## Materials and methods

This quasi-experimental study was conducted at the Dermatology Department of Combined Military Hospital Kohat, a tertiary healthcare setup in Kohat, Pakistan, between April 16, 2024 and October 16, 2024, over six months. Approval was obtained from the Institutional Ethical Review Committee of the hospital (Ethical Committee Approval Number E-2005/A/158), and informed, explicit consent was obtained from all the volunteers. A total of 122 female patients suffering from melasma for the past year, having a minimum modified Melasma Area Severity Index (mMASI) of ≥06, were included in the study.

The sample size was calculated using the WHO calculator with the power of the study at 95%, confidence level of 95%, proportion in Group 1 (P1) at 56%, and proportion in Group 2 (P2) at 90% [9,10]. The sample size turned out to be 78; however, due to the higher influx of melasma patients in our institute, the number of enrolled patients was increased to 110 to enhance the strength of the study.

Volunteer female patients, both married and unmarried, aged 18-55, who had been presenting with melasma for the last 12 months, had not received any topical or oral medications for melasma in the previous six months, and were willing to attend follow-up in the outpatient department, were included in the study. Since the majority of our patients reporting to our institute for seeking medical attention for melasma are females, this study was specifically directed and conducted on the female population.

Individuals with a history of any other dermatological conditions, such as cutaneous infections, discoid lupus erythematosus, a positive pregnancy test, history of lactation, use of oral contraceptives, and anaemia, and those unwilling to be photographed were excluded from the research. The patients were assigned to two groups (Group A and Group B) using a non-probability consecutive sampling technique. Randomisation was performed for the 110 volunteer patients in a 1:1 ratio for Group A and Group B, through which each incoming patient was assigned to the alternate group. In Group A (intralesional TA MT), 55 patients were included, while in Group B (AA MT), 55 patients were included. Group A received fortnightly intralesional pure TA (100 mg/ml), injected 1 cm apart via a sterile insulin syringe until the appearance of blanchable papules, while in Group B, intralesional injections of AA (L-Ascorbic Acid 20% in a 10mL vial) were administered 1 cm apart via a sterile insulin syringe until the appearance of blanchable papules. A total of six MT sessions were administered in both groups over 12 weeks. Both groups were also given a daily night-time topical Kligman regimen-based cream (Melakut© by Crystolite Pharma), along with sun protection in the form of a broad-spectrum sun protection factor 60 cream. All patients were educated in a 15-minute interactive session by specially trained dermatologists to apprise them of sun protection measures and to avoid any other home-based or commercially available topical creams.

Both groups were assessed at baseline, at the end of treatment, and at the 12-week follow-up using the mMASI score, digital photographs, and patient satisfaction questionnaires. The mMASI score was calculated by evaluating the area (A) and darkness (D) of melasma by dividing the face into four regions: forehead, left malar, right malar, and chin. The area of involvement (A) was rated from 0 to 6, with 0 indicating absence, 1 indicating up to 10% involvement, 2 (10-30%), 3 (30-50%), 4 (50-70%), 5 (70-90%), and 6 indicating 90-100% involvement. Similarly, the darkness (D) of melasma was rated from 0 to 4 (0 = absent, 1 = slight, 2 = mild, 3 = marked, 4 = maximum darkness). The total mMASI score ranged from 0 (clear) to 24 (most severe melasma), and the formula used to calculate the mMASI score was as follows: mMASI = 0.3 × A × D (forehead) + 0.3 × A × D (left malar) + 0.3 × A × D (right malar) + 0.1 × A × D (chin) [11].

Baseline demographics, such as age, duration, and pattern of melasma, and marital status were recorded. Safety and adverse effects were monitored and documented throughout the study period, and statistical analysis was performed to compare the efficacy, patient satisfaction, and side effect profiles of both treatment modalities.

Data analysis was performed using the IBM SPSS Statistics for Windows, Version 23.0 (released 2015, IBM Corp., Armonk, NY). For quantitative variables, mean and standard deviation were calculated, while for qualitative variables, percentage and frequency were noted. The chi-square test was used to compare the efficacy of the treatment modalities in both cohort groups, and a p-value of ≤0.05 was considered statistically significant.

## Results

This study included a total of 110 individuals aged between 18 and 55 years, with a mean age of 32.88 ± 6.23 years. Patients in Group A had a mean age of 34.4 ± 5.1 years, while those in Group B had a mean age of 32.8 ± 4.5 years. Around two-thirds (n = 79, 71.8%) of the cohort were in the age bracket of 18-35 years. Table 1 summarises the demographic characteristics of patients from both groups in terms of age, baseline mMASI scores, type of melasma, and duration of melasma.

**Table 1:** Demographic characteristics of patients in both groups, including age, baseline modified Melasma Area Severity Index (mMASI) scores, type of melasma, and duration.

Demographic characteristics	Group A (n = 55)	Group B (n = 55)	p-value
Age (years), mean ± SD	34.4 ± 5.1	32.8 ± 4.5	-
Type of melasma	Dermal: 30%	Dermal: 28%	-
	Mixed Type: 70%	Mixed Type: 72%	-
Baseline mMASI score, mean ± SD	17.3 ± 3.2	16.4 ± 3.5	0.521
Duration of melasma (months), mean ± SD	23.4 ± 6.3	24.2 ± 5.8	0.167

**Figure 1:**
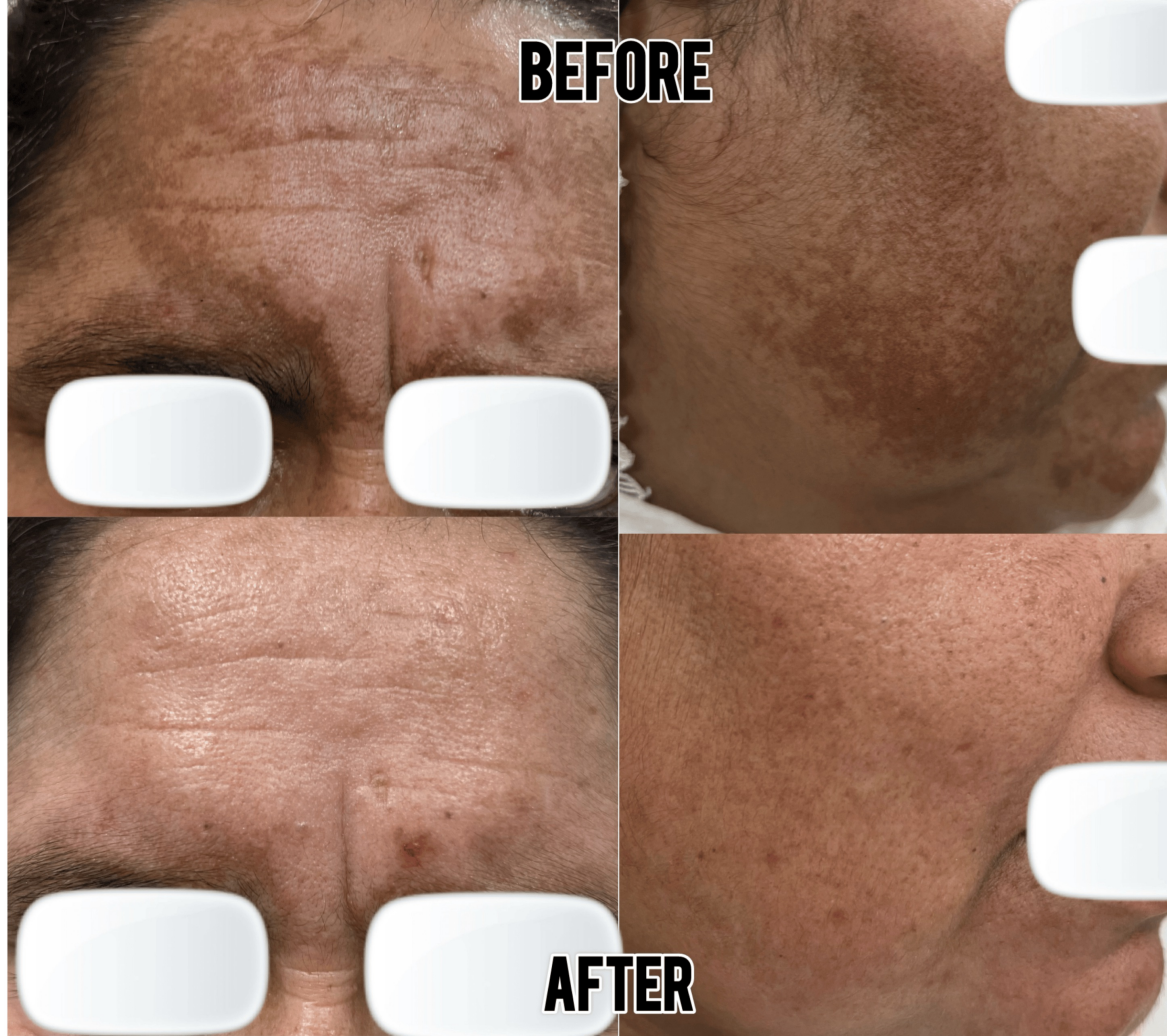
Treatment clinical presentation of a patient undergoing tranexamic acid mesotherapy, showing baseline melasma lesions.

**Figure 2:**
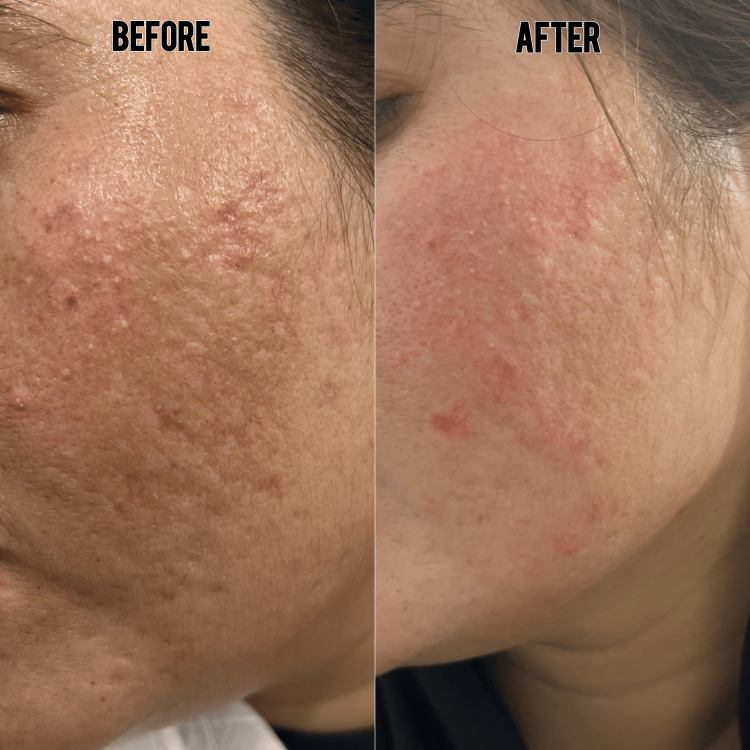
Treatment response after three sessions of tranexamic acid mesotherapy, demonstrating visible improvement in pigmentation alongside the Kligman regimen.

The comparison of mMASI scores for Groups A and B indicates improvement in the condition of patients over 16 weeks with both types of MT, as shown in Table 2 and Figures 1-2, respectively. The pretreatment mean MASI scores in both MT groups were comparable; however, TA MT showed substantially greater improvement in mMASI scores compared to AA MT.

**Table 2:** Comparative improvement in the modified Melasma Area Severity Index (mMASI) scores in Group A (tranexamic acid mesotherapy) and Group B (ascorbic acid mesotherapy) over a 16-week treatment period.

Duration of treatment	Type of treatment	mMASI score			p-value
		Minimum	Maximum	Mean	
Baseline	Tranexamic acid mesotherapy	6.4	8.1	7.2	-
	Ascorbic acid mesotherapy	6.3	7.9	7.1	
4 weeks	Tranexamic acid mesotherapy	5.1	7.2	6.1	0.001
	Ascorbic acid mesotherapy	5.9	7.6	6.75	
8 weeks	Tranexamic acid mesotherapy	4.4	5.7	5.05	0.001
	Ascorbic acid mesotherapy	5.9	7.4	6.65	
16 weeks	Tranexamic acid mesotherapy	3.3	5.6	4.45	0.001
	Ascorbic acid mesotherapy	5.8	6.7	6.25	

The adverse effects of both modalities are shown in Table 3. Although there was no statistically significant difference in the frequency of adverse events between the two treatments, the incidence of irritation was slightly higher in the AA MT group compared to the TA group. Most of the adverse events in our patients were of limited duration and severity in both groups, and these adverse effects did not impact the treatment adherence or require any intervention.

**Table 3:** Adverse effects observed in both treatment groups, including the frequency of erythema and irritation.

Adverse effects	Tranexamic acid mesotherapy	Ascorbic acid mesotherapy
Number of participants	55	55
None	87.5%	85.5%
Erythema	7.3%	6.5%
Irritation	3.2%	6.0 %
Erythema (along with irritation)	2%	2.0%

## Discussion

Melasma is characterised by dark pigmentation of the face. Its underlying pathophysiology is unclear, although various factors such as hormonal influences, sun exposure, certain medications, and family history and predisposition have been implicated [4,5,6]. Treatment strategies mainly focus on removing triggering factors and controlling the disease, as a definitive and permanent cure is still being sought.

Our research focuses on the comparative efficacy of two relatively novel approaches in melasma. Both TA and AA MT, used in variable concentrations and treatment frequencies, have been reported in multiple studies across the globe with promising, albeit equally variable, outcomes [7,8].

The efficacy of oral TA has also been supported by several studies in the management of melasma [12,13]. Its effectiveness has been linked to reduced tyrosinase activity, lower melanin levels, and decreased expression of tyrosine-related proteins 1 and 2 - two important mediators in melanin production [14]. However, using TA in MT form may improve compliance, as treatment is given fortnightly rather than daily. Studies have also reported higher efficacy of TA compared to other commonly used agents such as hydroquinone. For example, a study comparing 5% TA cream with 2% hydroquinone cream found a higher rate of satisfaction in the TA group, although the difference in MASI scores was not statistically significant. TA was also better tolerated, with fewer cases of erythema and skin irritation than hydroquinone [15].

Similarly, the use of AA creams and serums has been favorably reported by various studies in different pigmentary conditions, including melasma [10,14].

Our study provides insight into the comparative efficacy of TA and AA MT in the SOC population, where the available data remain limited. Dermatologists in SOC-dominant countries are often working in resource-poor settings where patients have low purchasing power. TA may offer a more affordable treatment option than vitamin C, which is comparatively more expensive. Since the side effect profiles of both compounds are similar, MT with TA appears to be a more attractive option.

The mean age of patients in our cohort is 32.88 ± 6.23 years, which is comparable to previous observations and closely matches findings from another South Asian study [16]. The efficacy of TA MT in that study was reported to be 44.14%, whereas in our study, around 70% of the patients showed a substantial reduction in mMASI scores with TA MT. This difference can be explained by the fact that the earlier study only compared TA MT with topical 4% hydroquinone, while our study administered the Kligman regimen in both groups.

MT is an effective drug delivery mechanism in dermatological settings, with the added benefit of avoiding various adverse events that might occur with oral or even topical administration of medications. Previous studies have reported enhanced penetration and improved efficacy when TA was delivered via MT [17]. Our study combined the efficient delivery of MT with already proven effective agents. While improved outcomes were expected due to the delivery method, our focus was also on examining whether a cheaper agent like TA could produce better results than a more expensive one like AA. We found that, despite being cheaper, MT with TA was also substantially more effective than AA. A considerable reduction in the MASI was noted in the TA group compared to the AA group after fortnightly MT sessions.

The administration of TA via MT also avoids potential side effects associated with oral intake. A study by Wu et al. reported that 5.4% of patients experienced gastrointestinal discomfort, while 8.1% reported hypomenorrhea [18]. Apart from a marginally higher incidence of erythema and irritation with AA MT, we did not observe any notable difference in the side effect profiles of the two treatment modalities.

The limitations of our study include the relatively small sample size. We also did not monitor the patients for the recurrence of melasma. Given the high recurrence rate of this condition, this is an important limitation. However, we are currently working on a follow-up project to evaluate the long-term outcomes of both treatments. The use of non-probability consecutive sampling and alternating assignment (not true randomisation) introduces a risk of allocation bias. True randomisation with allocation concealment in future trials to enhance internal validity is suggested. The lack of blinding of outcome assessors (MASI scoring and patient satisfaction) may have introduced assessment bias. As our study was conducted in the SOC population and focused on comparing the efficacy of TA and AA, we did not perform a direct comparison of these agents in fair-skinned individuals. Further large-scale, prospective interventional trials are needed to address these gaps.

## Conclusions

Our research concludes that TA MT may serve as an effective adjunct therapeutic option in the treatment of melasma, alongside well-established modalities like the Kligman regimen, in female patients with SOC. Our study limitations, such as a small sample size and shorter follow-up duration, should be taken into consideration. TA MT is not only more effective than AA MT in treating melasma, but also substantially more affordable and potentially safer, with fewer chances of irritation in this population. Dermatologists should consider endorsing TA MT as part of melasma treatment alongside the Kligman regimen, as this approach may offer improved therapeutic outcomes while reducing the economic burden on patients with SOC. Further large-scale, multicenter, and longer-term follow-up studies are warranted to evaluate recurrence rates and durability of response.
